# Effects of Sulforaphane Treatment on Skeletal Muscle from Exhaustive Exercise-Induced Inflammation and Oxidative Stress Through the Nrf2/HO-1 Signaling Pathway

**DOI:** 10.3390/antiox14020210

**Published:** 2025-02-12

**Authors:** Ruheea Taskin Ruhee, Sihui Ma, Katsuhiko Suzuki

**Affiliations:** 1 Japan Society for the Promotion of Sciences, Chiyoda Ku 102-0083, Tokyo, Japan; 2 Faculty of Human Sciences, Waseda University, Tokorozawa 359-1192, Japan; masihui@toki.waseda.jp; 3 Faculty of Sport Sciences, Waseda University, Tokorozawa 359-1192, Japan

**Keywords:** exercise, skeletal muscle, inflammation, oxidative stress

## Abstract

Skeletal muscle is primarily involved in exercise performance and health promotion. Sulforaphane (SFN) is a naturally occurring isothiocyanate that indirectly activates the transcription factor Nrf2 (nuclear factor erythroid 2-related factor 2), thus inducing the expression of Nrf2 target genes, including antioxidant enzymes. This study aimed to identify the effects of a single dose of SFN administration on exhaustive exercise-induced inflammation and oxidative stress in skeletal muscle tissue and elucidate the underlying mechanisms. Thirty-six mice were divided into four groups: control, SFN, exercise (Ex), and SFN + Ex. The SFN group and SFN + Ex group received SFN orally (50 mg/kg body weight) 2 h before the running test. Exercise significantly reduced plasma glucose levels, while the SFN-treated group exhibited a smaller reduction. Acute exhaustive exercise increased the expression of pro-inflammatory cytokines in muscle tissue, while the SFN + Ex group exhibited significantly reduced expression of pro-inflammatory cytokines. The gene expression of Nrf2 and its target enzymes, including heme oxygenase (HO)-1, superoxide dismutase (SOD)-1, catalase (CAT), and glutathione peroxidase (GPx)-1, was measured in the gastrocnemius and soleus muscle tissue. Compared with the Ex group, the SFN + Ex group showed upregulated expression of all these parameters, including Nrf2. SFN treatment reduced acute exhaustive exercise-induced oxidative stress and inflammation via activation of the Nrf2/HO-1 signaling pathway.

## 1. Introduction

Comprising approximately 40% of total body mass and 50% of total protein, skeletal muscles play a pivotal role in the body’s movement and energy metabolism [1]. The balance between protein synthesis and degradation in skeletal muscle is regulated via diverse molecular mechanisms, which also contribute to the maintenance of muscle mass. Furthermore, skeletal muscle mass ensures equilibrium condition between hypertrophic and atrophic signals. Depending on the intensity of exercise or training, skeletal muscles may undergo hypertrophy with either an increase in muscle endurance or size without accompanying changes in muscle strength [2,3]. The functional adaptations of muscle fibers are largely dependent on endurance capacity and strength training, both of which also alter the enzymatic profiles of muscle fibers [4,5,6].

Skeletal muscle fibers can be classified into slow-twitch (type I) and fast-twitch (type II) fibers. The soleus muscle is predominantly composed of slow-twitch fibers, exhibiting a red appearance due to its high myoglobin and capillary content. In contrast, the gastrocnemius muscle consists mainly of fast-twitch fibers and appears white [7]. The soleus muscle possesses a higher oxidative capacity and plays a crucial role in endurance exercise, while the gastrocnemius has lower aerobic endurance capacity and assumes a secondary role [8,9]. Endurance training enhances the oxidative capacity of both the soleus and gastrocnemius muscles and increases mitochondrial content in skeletal muscle without altering the fiber type ratio [10]. Mitochondrial health is maintained through fission, fusion, and mitophagy, which are promoted by endurance training [11,12,13]. Endurance athletes exhibit a higher proportion of slow-twitch fibers in their trained musculature, whereas sprinters’ muscles are primarily composed of fast-twitch fibers [10].

Exercise-induced muscle contraction activates various signaling pathways, including mitogen-activated protein kinase (MAPK), protein kinase C (PKC), nuclear factor kappa B (NF-κB), AMP-dependent protein kinase (AMPK), and Nrf2, which integrate stress and contraction-related signals [14,15]. Contractile activity increases reactive oxygen species (ROS) production in skeletal muscle due to elevated oxygen consumption [16]. When ROS levels exceed the antioxidant defense capacity, oxidative stress occurs, leading to disrupted redox signaling and control [17]. Strenuous exercise enhances blood flow to skeletal muscle but reduces perfusion to the liver, kidney, and testes [18]. Different types and intensities of exercise can increase oxidative stress markers in skeletal muscle cells [19], with prolonged acute exercise of concentric nature causing mitochondrial dysfunction in the working skeletal muscles [20]. Excessive ROS production in contracting skeletal muscle contributes to muscle fatigue [21].

The endogenous antioxidant system, comprising both enzymatic and non-enzymatic components, maintains the balance between oxidants and antioxidants. Superoxide dismutase (SOD), catalase (CAT), and glutathione peroxidase (GPx) serve as the first line of enzymatic antioxidant defense [22,23]. SOD catalyzes the conversion of superoxide anion to hydrogen peroxide, which is then neutralized by CAT and GPx to form water and oxygen [24].

Cytokines are regulatory proteins actively involved in the immune response and exercise adaptation [25]. They are rapidly synthesized in response to stressors or stimuli, mediating signal transduction through multiple transcription factors [26,27]. Anti-inflammatory cytokines, i.e., interleukin (IL)-4, IL-10, and IL-13, inhibit the production of pro-inflammatory cytokines, including tumor necrosis factor (TNF)-α, IL-6, and IL-1β, to protect exercise-induced muscle damage [28]. Endurance exercise increases oxygen consumption and free radical damage, leading to elevated pro-inflammatory cytokines and neutrophil accumulation in skeletal muscle [25,29,30].

**Figure 1:**
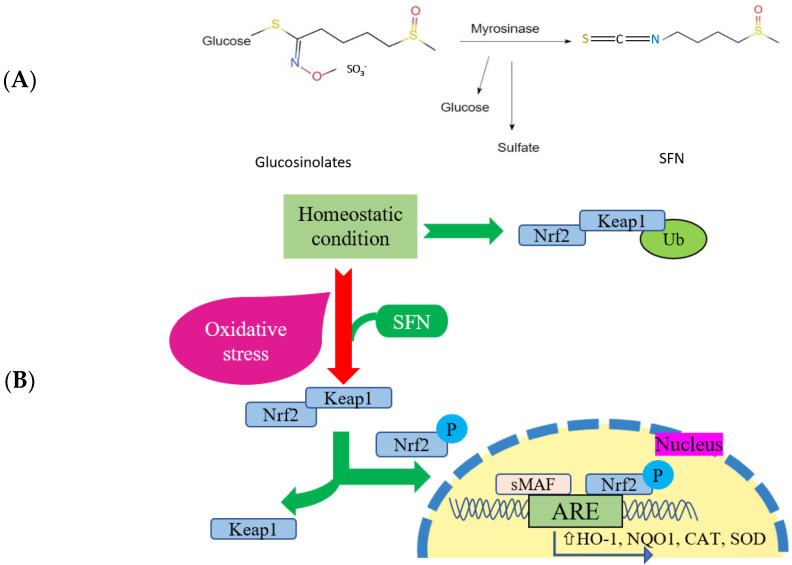
Chemical reaction and structure of SFN (**A**); interaction between Nrf2 and downstream antioxidant enzymes (**B**) (adapted from [55]).

Sulforaphane (SFN) is a hydrolysis product of glucosinolates (GSLs) [31], a biologically active phytochemical derived from broccoli, and belongs to the isothiocyanate family [32,33,34,35]. It is a thermosensitive molecule with lipophilic properties [36] and interacts with major transcription factors, such as Nrf2 and NF-κB [37]. SFN is primarily known for its tumor-suppressing properties and indirect antioxidant effects, including Nrf2 translocation and accumulation in [38] the nucleus under stressed conditions [39]. Activated Nrf2 also induces phase 2 detoxification enzymes (heme oxygenase: HO-1, glutathione S-transferase, NAD(P)H: Quinone oxidoreductase type 1: NQO1, UDP-glucuronosyltransferase: UGT, and sulfotransferase: SULT) [40], which detoxify toxic molecules and ROS, reducing the risk of cancer and degenerative diseases associated with oxidative stress and inflammation (Figure 1) [41,42,43,44]. Numerous in vivo and in vitro studies reported the mechanisms through which SFN mediates oxidative stress and inflammation through the modulation of major transcription factors [45,46,47,48,49,50,51,52,53,54].

This study aimed to investigate the potential protective effects of a single dose of SFN on exhaustive exercise-induced inflammation and oxidative stress in skeletal muscle, based on our previous findings of reduced plasma lactate dehydrogenase (LDH) in the SFN-treated exercise group [47]. We found that plasma LDH was reduced in the exercise group of SFN. Since LDH is also a muscle damage marker, we hypothesized that a single dose of SFN may potentially protect exercise-induced inflammation and oxidative stress in skeletal muscle. The dose used in this study was selected based on previously published articles [56,57].

## 2. Materials and Methods

### 2.1. Animal Maintenance and Diet

Male C57BL/6 mice (n = 36, seven weeks old) were purchased from the Takasugi Experimental Animals Supply (Kasukabe, Japan) and kept in a temperature- and humidity-controlled room for two weeks. The mice were randomly divided into four groups: sedentary control (CON, n = 9), SFN (n = 9), exercise (Ex, n = 9), and SFN + Ex (n = 9). The experimental procedures of this study followed the Guiding Principles for the Care and Use of Animals in the Academic Research Ethical Review Committee of Waseda University and were approved (2019-A109).

### 2.2. Acute Exhaustive Exercise and SFN Ingestion Protocol

**Figure 2:**
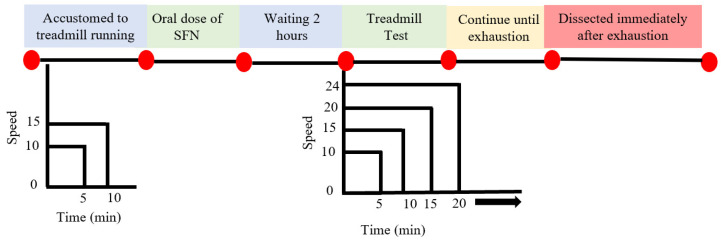
Experimental design and exhaustive exercise protocol.

All mice (including sedentary) were accustomed to running on a motorized treadmill (Natsume, Kyoto, Japan) at 15 m/min for 10 min (0% incline) one week before the exhaustive running exercise. On the day of the experiment, SFN (LKT Laboratories, St. Paul, MN, USA, product code S8044) was administered orally (50 mg/kg body weight), solubilized in 150 µL of distilled water, whereas an equivalent volume of distilled water was given to the control group. After two hours of gavage feeding, the uphill treadmill running (7% incline) was started. The Ex and SFN + Ex groups of mice were subjected to run at 15 m/min for 10 min, followed by 20 m/min for 10 min, and finally 24 m/min until exhaustion (Figure 2). The state of exhaustion was defined as the inability to continue running despite tapping on the back of the mouse several times or repositioning on the treadmill using the hand [58,59]. Running time was recorded for exercised mice. Immediately after exhaustion, heparinized blood samples were collected from the abdominal aorta under isoflurane-induced mild anesthesia. Tissue and organs were collected and immediately frozen in liquid nitrogen. Blood samples were centrifuged at 1500× *g* for 10 min at 4 °C. All the samples were stored at − 80 °C until analysis.

### 2.3. Plasma Glucose Measurement

Plasma levels of glucose were measured by Koutou-Biken Co. (Tsukuba, Japan) using the hexokinase-G-6-PDH method.

### 2.4. Real-Time (RT) Quantitative Polymerase Chain Reaction (qPCR)

From the gastrocnemius and soleus muscle tissue, total RNA was extracted using the TRizol extraction reagent (Thermo Fisher, Rockford, IL, USA), according to the maker’s protocol. The purity and concentration of extracted RNA were measured using the NanoDrop system (NanoDrop Technologies, Wilmington, DE, USA) with a ratio of A260/280. Then, using the High-Capacity cDNA Reverse Transcription kit (Applied Biosystems, Foster City, CA, USA), the RNA was reverse transcribed to cDNA, according to the provided instructions. PCR was performed using the Fast 7500 real-time PCR system (Applied Biosystems, Foster City, CA, USA) and Fast SYBR Green PCR Master Mix (Applied Biosystems, Foster City, CA, USA). For all genes, the thermal profile consisted of one denaturing cycle at 95 °C for 10 min, followed by 40 cycles of denaturing at 95 °C for 3 s, and annealing and elongation at 60 °C for 15 min. For the housekeeping gene, we used 18S mRNA. Using the ΔΔCT method, all data were normalized and stated as a fold change relative to the values of the control group. The specific sequences of primers used for gene amplification are given in Table 1.

### 2.5. Statistical Analysis

All data were presented as the mean ± standard error (SE). To identify the effects of two independent variables on a dependent variable, a two-way analysis of variance (ANOVA) was performed. We evaluated the main effect of SFN administration and/or exercise, followed by Tukey’s post-hoc test to determine significant differences among the means of each group. Statistical significance was defined as *p* < 0.05, using IBM SPSS, V 25.0 (IBM Corp., Armonk, NY, USA).

## 3. Results

### 3.1. Effects of SFN Treatment on Endurance Capacity Test and Plasma Glucose Level

**Figure 3:**
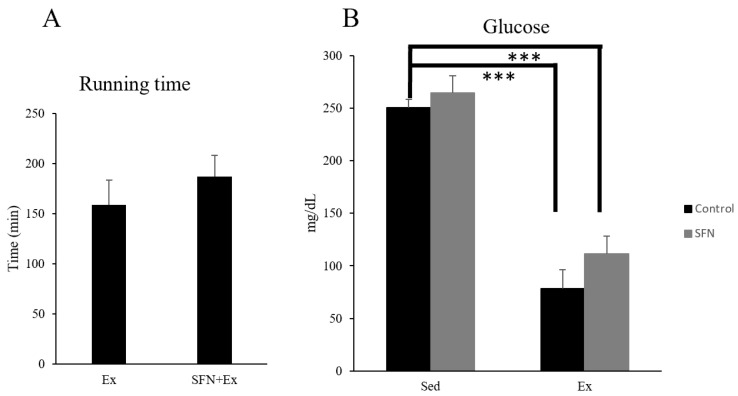
Running time comparison between groups (**A**). Plasma glucose level comparison between sedentary (SED) and exercise (Ex) groups with (Sulforaphane; SFN) or without (control) treatment (**B**). Data are presented as the mean ± SE. Statistical significance was defined as *p* < 0.05 and *** *p* < 0.001.

Running time for control group mice was 159 ± 25 min until exhaustion, while SFN-treated mice had a slightly longer running time of 187 ± 21 min, though the differences were not significant. Plasma glucose level was significantly reduced in both exercise groups compared to the sedentary control group. However, the SFN-treated exercise group showed a higher glucose level than the control exercise group (Figure 3).

### 3.2. Effect of SFN on Exercise-Induced Pro-Inflammatory Cytokines Gene Expression in Both Soleus and Gastrocnemius Muscles

**Figure 4:**
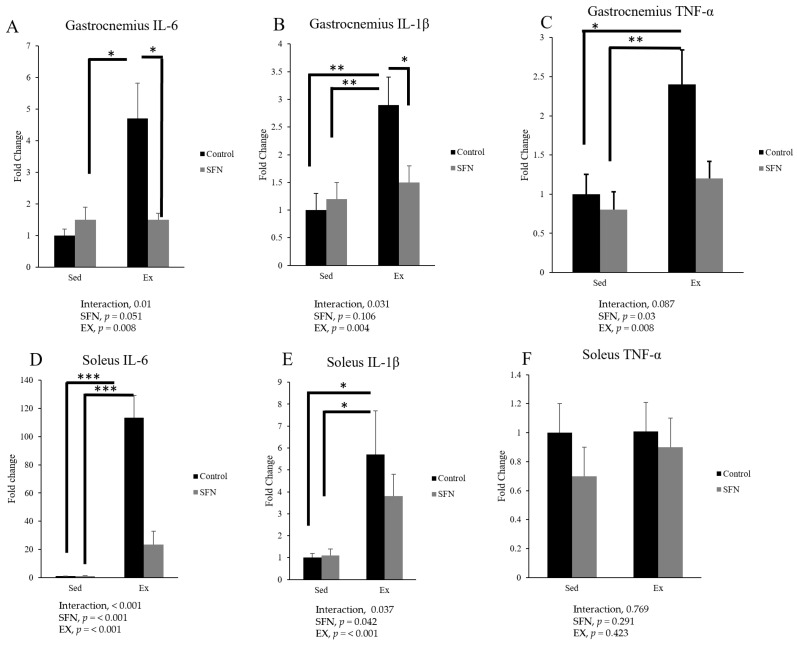
IL-6 mRNA, IL-1β, and TNF-α mRNA expression in the gastrocnemius (**A**–**C**) and soleus (**D**–**F**) muscles. The comparison was made between sedentary (Sed) and exercise (Ex) groups, with (sulforaphane, SFN) or without (control) supplementation. The interaction effect of SFN intervention and exercise was identified using two-way ANOVA, followed by Tukey’s post-hoc test to determine significant differences among the means of each group. Statistical significance was defined as *p* < 0.05. * *p* < 0.05, ** *p* <0.01, and *** *p* < 0.001. Data are presented as the mean ± SE.

We analyzed the gene expression of pro-inflammatory cytokines IL-6, IL-1β, and TNF-α mRNA in the gastrocnemius and soleus muscles using RT-qPCR to identify the effect of SFN on pro-inflammatory cytokine levels after endurance exercise (Figure 4). Results are expressed as relative differences from the sedentary control, which served as the baseline. IL-6 mRNA expression in both the gastrocnemius and soleus muscles fiber increased significantly in the Ex group, and SFN intervention significantly contributed to lower the expression of IL-6 mRNA. Such a result may indicate that IL-6 gene expression demonstrated a fiber type specificity and fiber structure. The gene expression of IL-1β increased 2.9-fold and 5.7-fold, respectively, in the Ex group for both gastrocnemius and soleus muscle tissues, and SFN + Ex group showed less expression in both muscle tissues. In the gastrocnemius muscle, a significantly reduced expression of TNF-α was observed in the SFN + Ex group compared to the non-intervention exercise group. However, in the soleus muscle, the difference was non-significant.

### 3.3. Effect of SFN on Antioxidant Defense Enzyme Gene Expression

**Figure 5:**
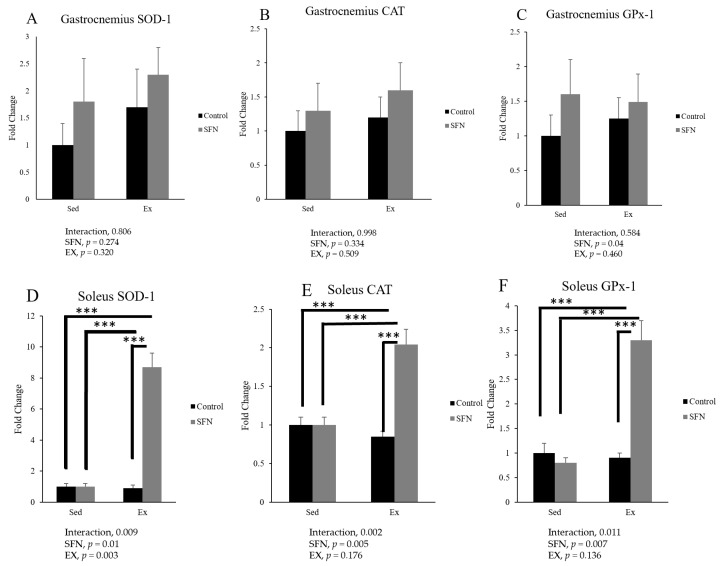
Comparison of superoxide dismutase (SOD)-1, catalase (CAT), and glutathione peroxidase (GPx)-1 mRNA expression in the gastrocnemius (**A**–**C**) and soleus (**D**–**F**) muscles between sedentary (Sed) and exercise (Ex) groups, with (sulforaphane, SFN) or without (control) supplementation. The interaction effect of SFN intervention and exercise was identified using two-way ANOVA, followed by Tukey’s post-hoc test to determine significant differences among the means of each group. Statistical significance was defined as *p* < 0.05 and *** *p* < 0.001. Data are presented as the mean ± SE.

SOD-1, CAT, and GPx-1 are also familiar as the first line of defense enzymes in the redox balance system against ROS. In the gastrocnemius muscle, the gene expression of SOD-1 was markedly increased in the SFN + Ex group compared to Ex and sedentary controls, while the gene expression of CAT and GPx-1 were slightly increased, but the differences were not significant (Figure 5). On the other hand, in the soleus muscle fibers, SOD-1, CAT, and GPx-1 were dramatically increased in the SFN+EX group and decreased in the Ex group compared to the sedentary control.

### 3.4. Effect of SFN on Nrf2/HO-1 Gene Expression in Skeletal Muscle Tissue

**Figure 6:**
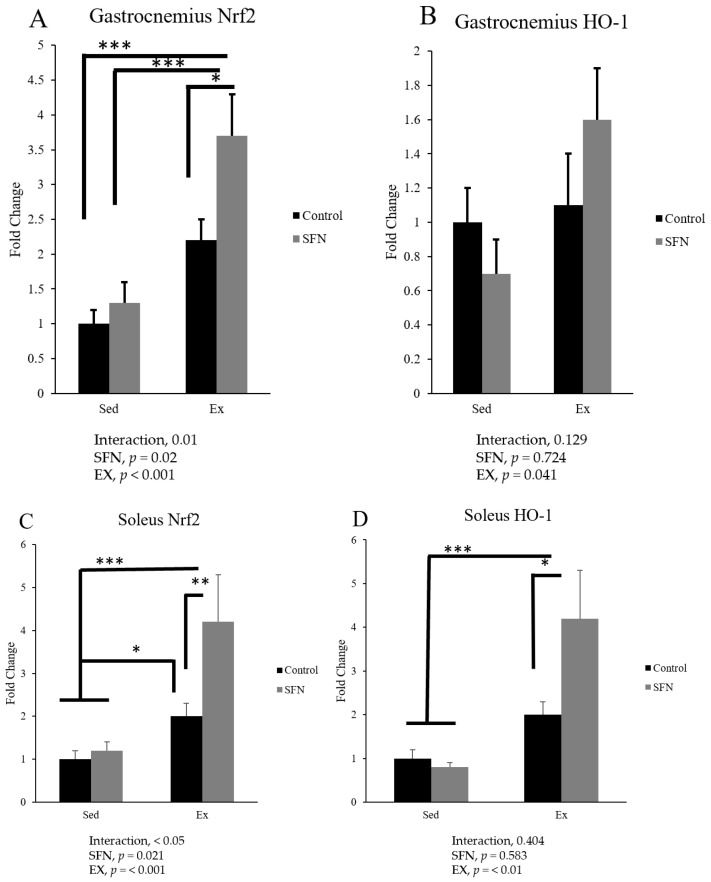
Comparison of nuclear factor E2-related factor (Nrf2) mRNA and heme oxygenase (HO)-1 mRNA expression in the gastrocnemius (**A**,**B**) and soleus (**C**,**D**) muscles between sedentary (Sed) and exercise (Ex) groups, with (sulforaphane: SFN) or without (control) supplementation. The main effects of SFN intervention and exercise were identified using two-way ANOVA, followed by Tukey’s post-hoc test to determine significant differences among the means of each group. Statistical significance was defined as *p* < 0.05, * *p* < 0.05, ** *p* < 0.01, and *** *p* < 0.001. Data are presented as the mean ± SE.

Nrf2 is one of the major transcription factors controlling the antioxidant defense system in both types of skeletal muscle fibers, and the SFN + Ex group significantly increased Nrf2 gene expression compared to the Ex and control groups (Figure 6). In the gastrocnemius muscle, phase 2 enzyme HO-1 expression increased by 1.8-fold in the SFN + Ex group compared to sedentary control, and in the soleus muscle, the expression increased by 4.2-fold in the SFN + Ex group compared to the Ex or sedentary control groups.

## 4. Discussion

In this experiment, we investigated the effects of SFN administration on exercise-induced muscle inflammation and alterations in antioxidant enzymes expression via the Nrf2/HO-1 signaling pathway. Combining the findings from our previous study on the protective effects of SFN against exercise-induced organ damage [47], we hypothesized that a single dose of SFN can minimize inflammation and oxidative stress in tissues actively involved during exercise, such as skeletal muscle and liver, by modulating the expression of antioxidant defense enzymes through the Nrf2/HO-1 signaling pathway.

Acute strenuous exercise is generally more challenging than controlled exercise training, leading to increased production of ROS, cytokines, and chemokines, which are associated with muscle fatigue [60]. Following a marathon race, TNF-α and IL-1β levels increase, accompanied by a dramatic 100-fold rise in IL-6 immediately post-exercise [25,61]. Specifically, the IL-6 production rate is higher in the acute phase response post-exercise compared to any other cytokines. Using RT-PCR, IL-6 was measured in skeletal muscle cells after eccentric or concentric exercise [62]. IL-6 is locally produced in muscle due to macrophage or neutrophil infiltration at the inflammatory site, causing tissue damage [63]. In this study, IL-6 gene expression exhibited fiber-type specificity, with a 113-fold increase in the soleus muscle and a 4.7-fold increase in the gastrocnemius muscle. We demonstrated for the first time that pre-treatment with SFN significantly reduced IL-6 gene expression in both muscle tissue types, as shown in Figure 4. The level of IL-6 production is an important indicator reflecting the degree of inflammation, as IL-6 increases rapidly during acute inflammation and initiates a cascade of inflammatory responses [64].

We investigated that TNF-α and IL-1β gene expression increased with exercise and decreased with SFN in both skeletal muscle types. Bufored et al. reported that three hours of resistance exercise significantly elevated the mRNA expression of TNF-α and IL-1β in skeletal muscles. However, no significant increase in serum TNF-α and IL-1β protein was observed, suggesting that skeletal muscle may be the source of the mRNA expression for these cytokines, contributing to muscle fatigue [65]. NF-κB, an inducible protein transcription factor, may be activated after a single bout of endurance exercise [66]. In the presence of circulating pro-inflammatory cytokines, the inhibitory I-κB kinases (IKK) are phosphorylated and degraded, allowing the NF-κB dimer to translocate into the nucleus [67] and induce the production of pro-inflammatory cytokines [68]. SFN can inhibit the nuclear translocation of NF-κB in an Nrf2-dependent manner in skeletal muscle, reducing the expression of circulating pro-inflammatory cytokines, such as IL-6, IL-1β, and TNF-α [52]. Also, our results newly demonstrated that SFN reduced pro-inflammatory cytokine expression in skeletal muscle.

The body’s antioxidant defense system maintains homeostasis by converting ROS into less reactive species. Antioxidant enzyme (SOD, CAT, and GPx) activity is significantly reduced after strenuous exercise [69,70]. Excessive free radical production or insufficient antioxidant response can disrupt redox homeostasis, leading to altered gene expression patterns. Acute strenuous exercise may deplete antioxidants stored in contractile skeletal muscle, further enhancing muscle fatigue, oxidative stress, and inflammation [71]. However, the role of this depletion in adaptation remains unclear [72]. Moreover, it is uncertain whether frequent consumption of antioxidant supplements minimizes exercise-induced muscle fatigue, oxidative stress, and inflammation [73,74,75]. SFN administration improved running distance in exhaustive endurance tests by increasing Nrf2-mediated target gene activities, including the upregulation of CAT enzyme mRNA expression [54]. Another recently published study reported that three days of SFN intervention before high-intensity interval training (HIIT) improved muscle antioxidant capacity in response to acute exhaustive exercise by significantly increasing the mRNA expression of antioxidant enzymes SOD-2, CAT, and GPx-1 [49]. In our study, we noticed a significant increase in SOD-1, CAT, and GPx-1 enzyme expression levels in the soleus muscle in the SFN + Ex group compared to the sedentary control and Ex groups. Acute exhaustive exercise may impair the antioxidant response of soleus muscle [76] due to the excessive production of NOX (NADPH Oxidase) 2/NOX4 enzymes in the soleus muscle [77].

SFN is considered a powerful antioxidant that activates the Nrf2 transcription factor, thereby preventing oxidative stress and inflammation. Acute exhaustive exercise induces oxidative stress and inflammation, causing injury in several organs, including skeletal muscle, such as impairment of muscle function and delayed onset muscle soreness (DOMS) [48]. Here, Nrf2 gene expression increased in both the gastrocnemius and soleus muscle in the SFN + Ex group compared to the exercise and sedentary control groups. Previous studies have demonstrated that SFN protects skeletal muscle by activating Nrf2 and enhancing the expression and activity of phase II enzymes [46]. When stimulated by an oxidative substance, Nrf2 translocates from the cytoplasm to the nucleus, binding to the promoter region to release downstream target genes and mediate the transcriptional activities of antioxidant enzymes [78]. SFN-activated Nrf2 also enhances endurance capacity and mitochondrial biogenesis through increased activity of the phase 2 detoxification enzyme HO-1 [54]. This transcriptional activation of Nrf2 also reduces lactate dehydrogenase (LDH) activity and pro-inflammatory cytokine concentrations, as well as muscle fatigue [45,46,48,52,54]. Furthermore, Nrf2 activation can independently influence several metabolic regulators like AMPK and PPAR-δ (peroxisome proliferator-activated receptor-delta) [79], which also play a crucial role in energy and lipid metabolism by lowering oxidative stress [49]. Urno et al. investigated that Nrf2 contributes to the maintenance of skeletal muscle glycogen metabolism by regulating the levels of glycogen branching enzyme (Gbe1) [80], resulting in increased glucose uptake [79]. In addition, Nrf2 enhances the transcription of IL-6, which indirectly contributes to improved glucose uptake [81]. This may explain the observed increase in glucose levels (Figure 3B) in the SFN + Ex group, improving energy metabolism and contributing to the longer running distance. However, a single dose of SFN did not enhance endurance capacity significantly; further research is required to understand the mechanism through which SFN influences endurance capacity. These data suggest that SFN protects skeletal muscle from acute exhaustive exercise-induced oxidative stress and inflammation through the activation of the Nrf2/HO-1 signaling pathway and antioxidant enzymes.

SFN supplementation effects depend on the dose, duration, and bioavailability [82]. Antioxidant supplementation can have some beneficial effects, but it can also be toxic at improper dosages and durations. The long-term effects of SFN supplementation in individuals engaged in regular physical exercise are unpredictable, and it is challenging to determine whether SFN supplementation affects other antioxidant mechanisms. Our study was limited to measuring the mRNA expression of several cytokines and enzymes rather than enzyme activity, as we aimed to investigate the signaling transduction mechanism. Based on the results, we hypothesized that SFN might act differently on specific muscle fiber types; therefore, we chose to measure mRNA expression in both the gastrocnemius and soleus muscles. However, we did not find any significant differences in mRNA expression between the two fiber types, except for IL-6. Regarding the endurance running test, the SFN + Ex group had a longer running time than the control exercise group, but the difference was not significant. Additionally, the plasma glucose level was relatively higher in the SFN + Ex group compared to the Ex group. Previous studies have reported that multiple doses of SFN administration may improve endurance capacity [54]. Since we used a single dose in this experiment, we chose a relatively high dose of SFN (non-lethal, within the safety range) compared to other published articles [46,52,54]. Moreover, the soleus muscle was too small to perform multiple analyses, and the limited volume of plasma collection restricted us to performing only biochemical analyses, which could be addressed using rats instead of mice in future studies. From the results of this study, we obtained a preliminary understanding that a single dose of SFN may downregulate exercise-induced inflammation and oxidative stress through the activation of the Nrf2/HO-1 signaling pathway and upregulation of antioxidant enzymes (Table 2). To overcome the limitations of this study, we are currently conducting cell culture experiments with muscle myoblasts to further extrapolate the results to animal or human studies.

## 5. Future Perspective and Conclusions

SFN is a phytochemical or plant-derived chemical substance that is nutrigenomically active but usually non-nutritive. To date, SFN has received considerable attention due to its ability to simultaneously regulate multiple cellular responses involved in various metabolic complications. The key transcription factors, Nrf2 and NF-κB, are responsible for redox modulation and inflammation-promoting gene expression. They act independently and cooperatively via cross-talk, which has yet to be discovered. The antioxidant, anti-inflammatory, and antimicrobial effects of SFN have shown promise in both in vivo and in vitro studies. A primary advantage of SFN is its high bioavailability, and it can be derived from the commonly produced broccoli sprout. However, the number of clinical trials on SFN is limited and specific, making it difficult to generalize its benefits. In the field of sports sciences, research on SFN is too limited to recommend it to athletes as a regular supplement. Perhaps, future research will focus more on its potential effects on athletic performance to resolve the current uncertainties.

To our knowledge, we are the first to report the effects of a single dose of SFN on exhaustive exercise-induced muscle inflammation and the underlying mechanisms. SFN is a potential activator of Nrf2 that concomitantly induces the expression of Nrf2 target genes. In addition to that, SFN enhanced antioxidant enzymes gene expression, along with a reduction in the expression of exercise-induced inflammatory cytokines. The amount of SFN we used in this experiment is quite high and may not be readily achievable through a normal diet. Therefore, further research is required to clarify the long-term effects of smaller doses of SFN on human health.

## Figures and Tables

**Table 1:** Specific primer sequences for RT-qPCR.

Target Gene	Accession Number (Size, bp)	Forward (5′ → 3′)	Reverse (5′ → 3′)
*Rn18s*	NM_011296 (127)	TTCTGGCCAACGGTCTAGACAAC	CCAGTGGTCTTGGTGTGCTGA
*Il6*	NM_031168 (76)	TAGTCCTTCCTACCCCAATTTCC	TTGGTCCTTAGCCACTCCTTC
*Tnf*α	NM_013693.1 (102)	CCTCCCTCTCATCAGTTCTA	ACTTGGTGGTTTGCTACGAC
*Il1β*	NM_008361.4 (183)	GGGCCTCAAAGGAAAGAATC	TTGCTTGGGATCCACACTCT
*Sod1*	NM_011434 (661)	GAGACCTGGGCAATGTGACT	GTTTACTGCGCAATCCCAAT
*Cat*	XM_028766902 (100)	CAGAGAGCGGATTCCTGAGAGA	CTTTGCCTTGGAGTATCTGGTGAT
*Gpx1*	NM_001329527.1 (751)	AGTACGGATTCCACGTTTGA	GGAACTTCTCAAAGTTCCAG
*Nfe212*	NM_010902 (100)	GAGTCGCTTGCCCTGGATATC	TCATGGCTGCCTCCAGAGAA
*Hmox1*	NM_010442 (175)	CACGCATATACCCGCTACCT	CCAGAGTGTTCATTCGAGCA

**Table 2:** Key findings from the results.

Measured Indicators	Findings (Main Interaction Between SFN and Ex)	
	Gastrocnemius	Soleus
IL-6 mRNA	Significant interaction found between the groups	Significant interaction found between the groups
IL-1β mRNA	Significant interaction found between the groups	Significant interaction found between the groups
TNF-α mRNA	Significant interaction found between the groups	Main interaction was not significant
SOD1 mRNA	Main interaction was not significant	Significant interaction found between the groups
CAT mRNA	Main interaction was not significant	Significant interaction found between the groups
GPx-1 mRNA	Main interaction was not significant	Significant interaction found between the groups
Nrf2 mRNA	Significant interaction found between the groups	Significant interaction found between the groups
HO-1 mRNA	Significant interaction found between the groups	Significant interaction found between the groups

## Data Availability

Data are presented within this article.
